# Cleansing efficacy of an auto-cleaning electronic toothbrushing device: a randomized-controlled crossover pilot study

**DOI:** 10.1007/s00784-020-03359-5

**Published:** 2020-06-06

**Authors:** Dagmar Schnabl, Vera Wiesmüller, Vera Hönlinger, Simon Wimmer, Emanuel Bruckmoser, Ines Kapferer-Seebacher

**Affiliations:** 1 Department of Operative and Prosthetic Dentistry, Medical University of Innsbruck, Anichstr. 35, 6020 Innsbruck, Austria; 2 Private Practice for Oral and Maxillofacial Surgery, 5020 Salzburg, Austria

**Keywords:** Auto-cleaning, Biofilm(s), Electric, Oral hygiene, Plaque index

## Abstract

**Objectives:**

To compare the cleansing efficacy of a representative “ten seconds” auto-cleaning device with that of uninstructed manual toothbrushing in a pilot study.

**Materials and methods:**

Twenty periodontally healthy probands refrained from oral hygiene for 3 days. Baseline full-mouth plaque scores (Rustogi Modified Navy Plaque Index, RMNPI) were assessed. After randomization, probands cleaned their teeth either with the auto-cleaning test device according to the manufacturer’s protocol or with a manual toothbrush. Plaque reduction was assessed by two aligned blinded investigators. After a 2-week recovery, the clinical investigation was repeated in a crossover design. The brushing pattern of the auto-cleaning device was analyzed in probands’ casts.

**Results:**

Full-mouth plaque reduction was 11.37 ± 3.70% for the auto-cleaning device and 31.39 ± 5.27% for manual toothbrushing (*p* < 0.0001). The investigation of the auto-cleaning device’s brushing pattern in dental casts revealed a positive relationship of bristle rows in contact with tooth surfaces and the cleansing efficacy in the respective areas. A maximum of 2/4 bristle rows were in contact with the tooth surfaces; in some areas, the bristles had no contact to the teeth.

**Conclusions:**

Uninstructed manual toothbrushing is superior to auto-cleaning. The alignment and density of the auto-cleaning device’s bristle rows need to be improved, and assorted sizes would be necessary to cover different jaw shapes.

**Clinical relevance:**

The auto-cleaning device has been developed to accommodate individuals with poor dexterity or compliance. To date, it is unable to provide sufficient plaque reduction due to an inappropriate bristle alignment and poor fit with diverse dental arches.

## Introduction

The regular removal of dental biofilm plays a key role in the maintenance of oral health. Tooth cleaning with a manual brush may well be effective in dependence of the user’s dexterity, the devoted time, and the applied brushing technique. There is moderate-quality evidence that powered toothbrushes provide a statistically significant benefit compared with manual toothbrushes with respect to plaque reduction and gingivitis in short- and long-term use [1, 2]. Instructed use of both manual and powered toothbrushes as well as of interdental devices is strongly advisable, in order to assure an efficient brushing technique for the respective toothbrush design and to prevent application of too much force or scrubbing [3]. Twice daily brushing for 2 min and a systematic pattern have been advised [2]. Although it is acknowledged how domestic oral health care should be performed, epidemiologic surveys point at a lack of efficient biofilm removal and awareness in the general population. In 16/20 European countries, 25–50% of teenagers brush their teeth less than twice a day [4]. Even children trained at school in group prophylaxis programs have low efficiency to adopt the toothbrushing recommendations given: Only 7.5% of the children brushed both inner and outer surfaces by the intended movements for at least 90% of the respective brushing time [5]. A recent study showed that even after performing oral hygiene to the best of one’s abilities, gingival margins showed persistent plaque at 69.48% ± 12.31% sites (mean ± SD) [6].

**Fig. 1:**
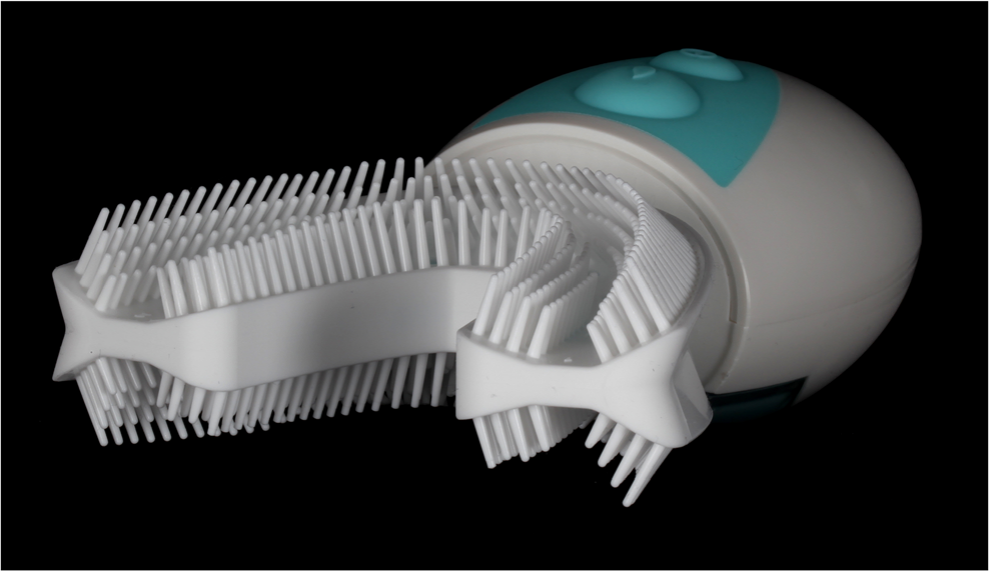
The auto-cleaning device *amabrush*®. The horseshoe-shaped biplane mouthpiece is mounted with four rows (two rows orally in the anterior region) of silicone bristles at the oral and vestibular side of the upper and lower jaw part. Bristles are aligned in 45° against the marginal gum in order to simulate the bass method

To facilitate tooth cleaning in persons with poor dexterity, impaired motor function, or just a lack of motivation, diverse manual and powered toothbrush designs have been developed. Within the past months, various auto-cleaning *U*-shaped toothbrushing devices have popped up on the (online) market promising “clean teeth” in a few seconds. To the authors’ knowledge, *amabrush*®, which was tested in the present study, was the first contemporary auto-cleaning device that was available on the European market. The horseshoe-shaped biplane mouthpiece is mounted with four rows (two rows orally in the anterior region) of silicone bristles at the oral and vestibular side of the upper and lower jaw part (Fig. 1). According to the manufacturer’s description, the bristles are aligned in 45° against the marginal gum in order to simulate the Bass method. The mouthpiece has to be attached to a rechargeable handpiece that contains a toothpaste pod from which, at button press, a defined amount of toothpaste sufficient for one brushing session is released. “More than 20,000 bristle oscillations per minute” are designated to cleanse the teeth within 10 s. Similar auto-cleaning devices available on the online market are, e.g., *Cartoon Blue-ray Whitening Teeth Brush®*, *Ultraschall Elektrische Zahnbürste Teeth Whitening Kit*®, *Automatic U Type Head Intelligent Wireless Charging Electric Toothbrush*®, or *Automatic Whitening Toothbrush*®.

The purpose of this randomized-controlled and single-blinded crossover pilot study was to compare the cleansing efficacy of the representative auto-cleaning device *amabrush*® with that of uninstructed manual toothbrushing. The null hypothesis was that there would be no difference in plaque reduction between the two brushing methods in randomly selected probands.

## Material and methods

The Ethics committee of the Medical University of Innsbruck, Austria, approved the study (ID AN 5123). The study was conducted in accordance with the 1964 Helsinki declaration and its later amendments. All subjects signed an informed written consent prior to the study enrollment.

### Study subjects

Twenty volunteers were recruited from the authors’ circle of acquaintances. Inclusion criteria were (1) age ≥ 14 years, (2) contractual capability, and (3) the presence of ≥ 5 teeth per quadrant. Exclusion criteria were (1) dental or medical students or professionals, (2) oral hygiene instructions prior to the study, (3) community periodontal index of treatment needs (CPITN) grade 3 or 4 [7], (4) pregnancy or breastfeeding, (5) systemic diseases or conditions that are associated with an increased risk of infection or necessitate concomitant antibiotic therapy with dental treatment, and (6) mental and behavioral disorders that impede (verbal) communication. Recruitment was performed from March 15 to April 14, 2019, and data collection was carried out from April 26 to May 13, 2019.

### Clinical intervention

The cleansing efficacy of brushing with the *amabrush*® versus manual toothbrushing was evaluated in a randomized-controlled, examiner-blinded, two-period crossover study. Each subject was asked to attend four appointments. At day one, the probands were informed about the study procedure; they signed an informed consent, and inclusion and exclusion criteria were proofed. After plaque disclosing (*2Tone*, Young, Earth City, Mo, USA), professional tooth cleaning was accomplished with an air-polishing device (*Airflow*® prophylaxis master and *Airflow*® Plus powder; both EMS, Nyon, CH), and, if appropriate, with sonic scalers and rubber cups with polishing paste (*Cleanic*®, Kerr, Bioggo, CH). Each proband was instructed to refrain from oral hygiene, including toothbrushing, the use of dental floss or other interdental cleaning devices, and the use of mouth rinses or chewing gum for 3 days. According to a computer-generated randomization (*Microsoft*® Office Excel), probands were allocated either to group 1, designated to start with using the *amabrush*®, or group 2, determined to start with manual toothbrushing. After 3 days of undisturbed biofilm accumulation, plaque was disclosed and scored by two blinded investigators (DS and WS) using the Rustogi Modified Navy Plaque Index (RMPN) [8] before (baseline) and after brushing with the assigned device. Probands of group 1 were assisted with using the *amabrush*® according to the manufacturer’s instructions. Only one size (model “Amabrush Version 1.0”) was available at the time the study was conducted. The mouthpiece was wetted and attached to the handpiece, which was loaded with the pod containing the “fresh” toothpaste. The toothpaste button was pressed to inject the toothpaste. After insertion of the mouthpiece and adjustment between the dental arches so as to ensure maximum fit, the start button was pressed. After 10 s, the brushing automatically stopped. The mouthpiece was removed and the probands were instructed to rinse with water. The RMNPI was assessed and teeth were air-polished. Probands of the group 2 were told to brush their teeth with a manual toothbrush (*Oral B Indicator Medium 35*®*,* Procter & Gamble UK, Weybridge, Surrey, UK) that had been dipped once into the same (liquid) “fresh” toothpaste, which had been poured into a cup. Toothbrushing was performed without instruction and in the absence of a mirror to ensure that the probands had no visual control of the disclosed plaque. The respective brushing method was recorded and the brushing time was stopped and chosen freely up to a maximum of 4 min. After rinsing with water, the RMNPI was assessed and air-polishing was performed. After a wash out phase of 11 days when the probands were practicing their usual oral hygiene procedures, they presented for the third visit (day 15). Again, plaque was disclosed and teeth were cleaned by air-polishing. After abolishing oral hygiene for 3 days, the fourth visit (day 18) unfolded in analogy to the second visit, with group 1 using the manual brush and group 2 using the auto-cleaning device.

Alginate impressions of both jaws were taken to obtain stone plaster casts for the evaluation of the size and shape of the dental arches and the investigation of the auto-cleaning device’s fit.

### Rustogi Modified Navy Plaque Index

The index divides buccal and lingual surfaces into nine areas (A to I) that are scored for the presence (score = 1) or absence (score = 2) of plaque. It assesses the amount of plaque on a whole-mouth basis (areas A–I), interdental basis (areas D and F), and the gingival margin basis (areas A–C). Third molars and deeply carious teeth were excluded from the evaluation, whereas teeth with fillings, inlays, onlays, or crowns were included. RMPNI is calculated as percentage of biofilm adhering sites to measured sites.

Examiner alignment and assessment was performed in five probands. The RMNPI was mutually assessed and agreed. Inter-examiner reliability was calculated with the Cohen’s kappa coefficient [9, 10] based on 9072 areas measured by both clinical investigators. Cohen’s kappa coefficient for RMPNI was *κ* = 0.768, reflecting a substantial inter-examiner reliability.

### Statistical analysis

Due to lack of previous investigations, the pilot study was done on five participants. The reduction of the mean plaque score (RMNPI) applying conventional toothbrushing was RMNPI = 30.81 ± 5.17%, and for the auto-cleaning device it was RMNPI = 13.08 ± 2.96%. Sample size calculation for dependent samples, a power of 90% and *α* = 0.05 revealed a sample size of three. Sample size was increased to 20 to improve the validity of the study.

For descriptive analysis and if not stated otherwise, mean and standard deviation are given. On a proband level, RMNPI values were calculated as the total number of tooth areas with plaque present divided by the total number of tooth areas scored (for 28 teeth, there was a total of 504 sites for the whole mouth, 112 sites for the interdental, and 168 sites for the marginal areas). The amounts of plaque reduction (pre-minus post-plaque removal amounts) were calculated and mean reduction in the whole-mouth plaque, interdental, and marginal plaque scores were compared between the two toothbrushing procedures by Wilcoxon signed-rank test. The significance level was set at *p* ≤ 0.05.

## Results

Twenty individuals (ten females and ten males; 19 Caucasians and one Asian) with a mean age of 26.25 ± 5.53 years (range 21–45) participated in this study. All participants were non-smokers. At baseline, 4 subjects had CPITN 0 and 16 displayed CPITN2.

For manual toothbrushing, the mean brushing time was 2.93 ± 0.91 min. Natural brushing technique was used by 11, classical Bass technique and red-to-white technique by four probands, and modified Bass technique by one individual.

### Plaque scores (Table 1)

After 3 days of plaque accumulation, full-mouth RMNPI was 42.81 ± 8.27% for the investigation of manual toothbrushing and 45.30 ± 14.63% for the auto-cleaning device (*p* > 0.05). Subgroup analyses of anterior and posterior teeth as well as buccal and lingual dental surfaces revealed no statistically significant differences for baseline plaque scores.

**Table 1:** **Plaque scores before and after cleaning.** Plaque was scored using the Rustogi Modified Navy Plaque Index (RMNPI) before (baseline) and after plaque removal. The index divides buccal and lingual surfaces into nine areas that are scored for the presence (score = 1) or absence (score = 0) of plaque. Plaque scores were calculated as the total number of tooth areas with plaque present divided by the total number of tooth areas scored. The amounts of plaque reduction were calculated: baseline minus after cleaning plaque score. Differences in plaque reduction were calculated for whole-mouth plaques scores, as well as buccal and lingual, and marginal and interdental plaque scores

	Baseline	After cleaning	Plaque reduction	*p* value**
Whole-mouth plaque scores (%)				
Manual toothbrush	42.81 ± 8.27	11.42 ± 6.19	31.39 ± 5.27	< 0.0001
Auto-cleaning device	45.30 ± 14.63	33.94 ± 13.18	11.37 ± 3.70	0.015
*p* value*	0.374	< 0.0001	< 0.0001	
Buccal plaque scores (%)				
Manual toothbrush	50.90 ± 11.06	7.85 ± 5.69	43.05 ± 9.00	< .0001
Auto-cleaning device	54.11 ± 19.25	42.33 ± 17.89	11.78 ± 6.62	0.052
*p* values*	0.482	< .0001	< .0001	
Lingual plaque scores (%)				
Manual toothbrush	35.07 ± 8.95	15.03 ± 9.55	20.04 ± 6.54	< .0001
Auto-cleaning device	36.46 ± 11.94	25.55 ± 9.76	10.91 ± 3.88	0.003
*p* values*	0.599	0.0006	< .0001	
Marginal plaque scores (%)				
Manual toothbrush	82.03 ± 13.26	25.08 ± 14.35	56.95 ± 11.34	< .0001
Auto-cleaning device	80.69 ± 19.38	65.40 ± 21.84	11.37 ± 3.70	0.024
*p* values*	0.796	< .0001	< .0001	
Interdental plaque scores (%)				
Manual toothbrush	46.06 ± 14.32	9.88 ± 8.39	36.18 ± 10.54	< .0001
Auto-cleaning device	52.48 ± 22.33	37.46 ± 19.10	15.02 ± 6.63	0.027
*p* values*	0.286	< .0001	< .0001	

*p values manual toothbrushing versus auto-cleaning device; **p values baseline versus after cleaning

**Fig. 2:**
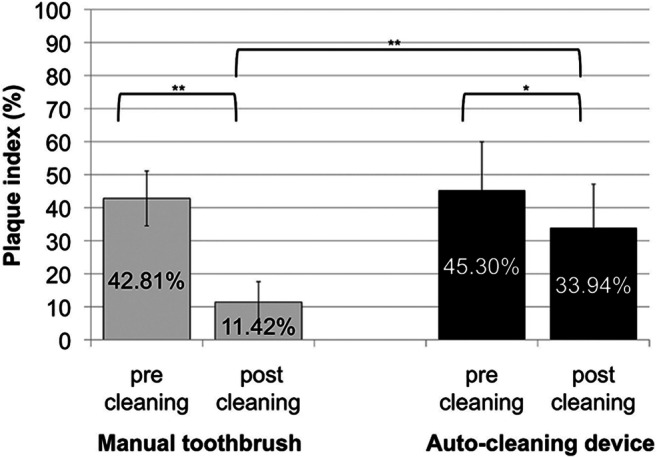
**Whole-mouth Rustogi Modified Navy Plaque Index (RMNPI) before and after plaque removal**. The RMNPI divides buccal and lingual surfaces into nine areas that are scored for the presence (score = 1) or absence (score = 0) of plaque. Plaque index in % was calculated as the total number of tooth areas with plaque present divided by the total number of tooth areas scored. Asterisks mark statistically significant differences (**p < 0.0001; *p < 0.05)

Immediately after brushing, statistically significant reductions in whole-mouth plaque scores were observed for manual toothbrushing (mean reduction of 31.39 ± 5.27%; *p* < 0.0001) as well as for the auto-cleaning device (reduction of 11.37 ± 3.70%; *p* = 0.015) (Table 1). For manual toothbrushing, there was no statistically significant correlation between brushing time and reduction of plaque index (*r*^2^ = 0.032; *p* = 0.451). Reduction of full-mouth RMNPI was significantly lower for the auto-cleaning device than for the manual toothbrush (*p* < 0.0001) (Fig. 2).

**Fig. 3:**
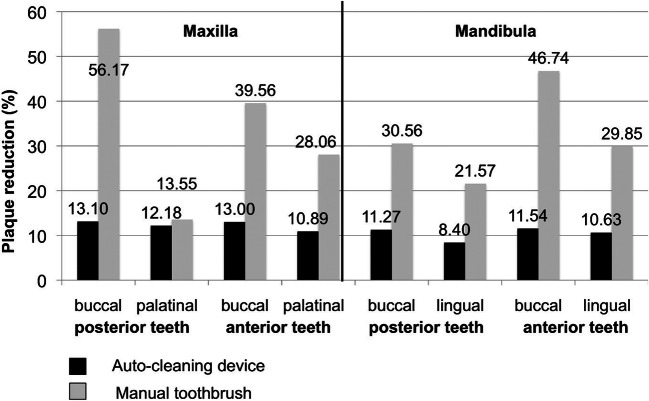
**Subgroup analysis of plaque reduction. **Whole-mouth Rustogi Modified Navy Plaque Index (RMNPI) was measured before and after plaque removal. Plaque index in percent was calculated as the total number of tooth areas with plaque present divided by the total number of tooth areas scored. Reduction of RMNPI before to after brushing is given as absolute value. At the palatal aspects of maxillary teeth, the auto-cleaning device brushed as efficiently as the manual toothbrush (*p* = 0.586). This was due to a lower efficiency of manual toothbrushing in this area. In all other areas, manual toothbrushing received a statistically significant higher plaque reduction than the auto-cleaning device (*p* < 0.0001)

Subgroup analyses for buccal and lingual, marginal, and interdental areas revealed statistically significant reductions of plaque scores for all areas with the manual toothbrush (*p* < 0.0001). This was also true for the auto-cleaning device with exception of the buccal surfaces, where no statistically significant reduction of plaque scores was attained (baseline RMNPI 54.11 ± 19.25%; post cleaning RMNPI 42.33 ± 17.89%; *p* = 0.052). In all subgroup analyses, manual toothbrushing achieved statistically significantly higher plaque reduction than the auto-cleaning device (*p* < 0.0001; Table 1). We further differentiated into anterior (canine to canine) and posterior teeth (molars and premolars) (Fig. 3). There was one region where the auto-cleaning device brushed as efficiently as the manual toothbrush. Mean plaque reduction on the palatal aspects of upper molars and premolars was 12.18 ± 6.96% for the auto-cleaning device and 13.55 ± 8.63% for the manual toothbrush (*p* = 0.586).

### Correlation analysis

Brushing efficacy of the auto-cleaning device was further analyzed with regard to the widths and lengths of the jaw arches. There were no statistically significant correlations between the brushing efficacy and the width or length of the jaws, neither for posterior nor for anterior regions, neither for buccal nor for oral tooth sites (data not shown).

**Fig. 4:**
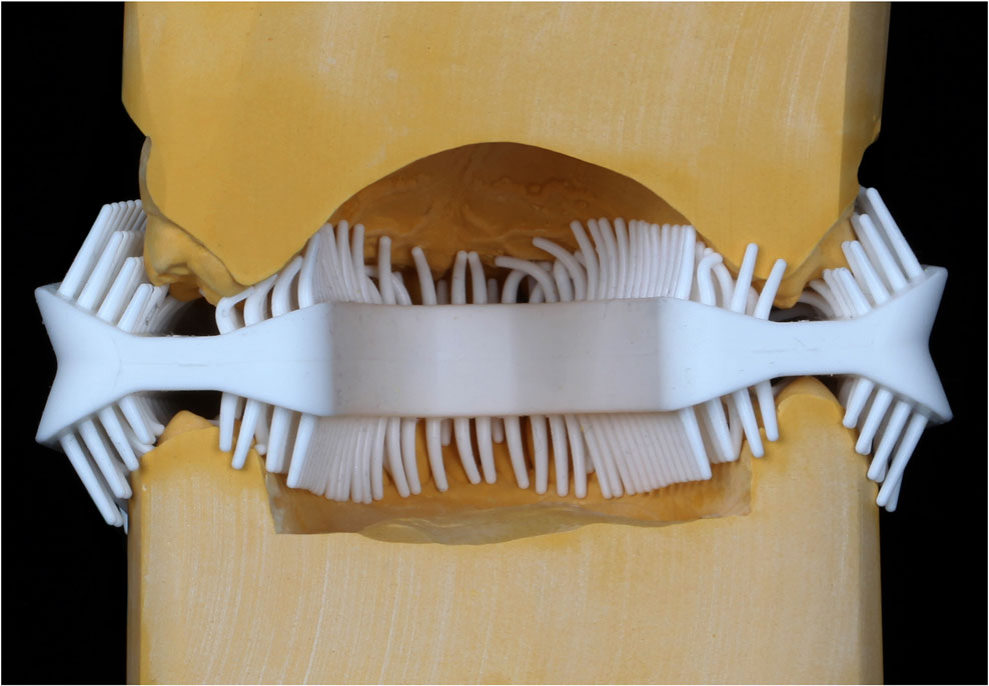
**Assessment of the quantity/intimacy of bristle contact to the tooth surfaces in casts**. The outer bristle rows are not in contact with the teeth. Instead, they touch the palatal/lingual gingiva or stand in distance to the tooth surfaces

We then investigated the number of bristle rows in intimate contact with the tooth surfaces. In none of the jaws or regions, the outer bristle row was in contact with the teeth. Instead, it was touching the palatal gingiva or stood in distance to the tooth surfaces during the cleansing process (Fig. 4). The most inner bristle row snapped off occlusally in small jaws. Then, we looked in detail on the buccal aspects; in 15/20 jaws, only one or none of the four bristle rows were in contact with the teeth leading to a significantly lower mean plaque reduction compared to jaws where two bristle rows were in contact (plaque reduction of 8.95 ± 8.84% and 20.67 ± 9.01%, respectively; *p* = 0.025). In none of the buccal areas, more than two bristle rows where involved in the cleaning process. Plaque reduction in posterior lingual/palatal regions was significantly higher if two or three bristle rows where in intimate contact with the tooth surfaces (17.82 ± 6.44%); if one or no bristle row was in contact, plaque reduction was low (7.56 ± 2.57%; *p* < 0.0001).

## Discussion

This clinical study was set up to test an auto-cleaning device that was designed to motivate and accommodate neglectful toothbrushers as well as individuals who suffer from an impaired motor function, and their caretakers. The prospect of “clean teeth within ten seconds” seems indeed tempting. When following the advice of brushing with a manual or electric toothbrush for at least 2 min [2], a fully dentate individual brushes each tooth for a mean of 4 s. In contrast to the conventional consecutive brushing of one tooth after another, the *amabrush*® fully covers both dental arches and is geared to simultaneously clean all tooth surfaces at once.

In the present study, the cleansing efficacy of the auto-cleaning device was clearly inferior to that of manual toothbrushing, and the null hypothesis was rejected. None of the individuals reached an equal or higher plaque reduction with the auto-cleaning device (range of plaque reduction 6 to 19%) compared to manual toothbrushing (range 22 to 44%). In subgroup analyses, there was one area where the auto-cleaning device was equally efficient in plaque reduction as the manual toothbrush. Mean plaque reduction on the palatal aspects of upper molars and premolars was 12.18 ± 6.96% for the auto-cleaning device and 13.55 ± 8.63% for the manual toothbrush (*p* = 0.586). Thus, lack of a statistically significant difference in this area was not due to a higher brushing efficacy of the auto-cleaning device but due to lower plaque reduction with the manual toothbrush compared to other regions (see Fig. 4).

We compared the cleansing efficacy of the auto-cleaning device with that of uninstructed manual toothbrushing in terms that no stipulations were imposed. In these days and age, everybody has had some oral hygiene instruction from an early age. Nevertheless, brushing efficacy is inferior in the general population. Deinzer et al. investigated the brushing behavior of probands instructed to perform toothbrushing to their best abilities [11]. They found that even though the total brushing time of the “to the best of one’s abilities” group exceeded that of the “common oral hygiene” group by more than a minute, the brushing efficacy did not exceed. The group supplemented their studies with an additional clinical investigation showing that efficacy of toothbrushing is not a matter of brushing time or technique but of establishing brushing systematics [12]. Therefore, the manual brushing time was not limited in our study. It amounted to a mean of almost 3 min, which is considerably longer than reported by other observational studies in uninstructed individuals, ranging from less than 1 min up to around two and a half minutes [13, 14], but reflects the brushing time reported by Deinzer et al. for probands brushing “to the best of one’s abilities” [11]. There was no statistically significant correlation between manual toothbrushing time and reduction of plaque index (*r*^2^ = 0.032; *p* = 0.451).

In order to create identical conditions for both tested brushing devices, the liquid toothpaste that comes with the *amabrush*® was also used for manual toothbrushing. By the use of a conventional (pasty and maybe more abrasive “real life”) dentifrice, manual toothbrushing might have been even more effective than it was using the watery toothpaste from the pod.

The RMNPI presents an elaborate and time-consuming index that is based on a dichotomous principle, which differentiates between the absence and presence of plaque in nine areas at buccal and lingual surfaces of the teeth. In contrast to other plaque indices, it allows analysis of interdental, marginal, and buccal/oral surfaces in the same way. Ordinal plaque indices like the commonly used Turesky-Modified Quigley-Hein Plaque Index (T-QHI) allow a grading of the plaque amount and might have yielded somewhat more differentiated results; however, it may pose considerable difficulties as appropriate descriptive analyses are missing and/or are difficult to translate to a daily clinic. A previous study comparing both, RMNPI and T-QHI, showed a strong correlation between indices for both pre- and post-brushing plaque scores [15]. The overall plaque reduction (31.39 ± 5.27%; *p* < 0.0001) by manual toothbrushing in our study is well within the range of 15 to 69% plaque reduction by uninstructed manual toothbrushing (duration 30 s up to 2 min, or unrestricted; assessed with either the T-QHI or the RNMPI) as found in a review/meta-analysis by Elkerbout et al. [16]. In interproximal sites, we found a mean plaque reduction of 36.18 ± 10.54; *p* < 0.0001 for customary manual toothbrushing. Other studies, for comparison, reported a range of 46 to 49% interdental plaque reduction (significance level *p* = 0.18 or *p* > 0.1, respectively) by use of a manual toothbrush [17, 18]. A potential selection bias by recruitment of potentially keen to do well probands from the authors’ set of acquaintances may possibly account for favorable manual toothbrushing results.

In the authors’ opinion, the development of auto-cleaning devices seems a gratifying approach to increase both the frequency and the efficacy of toothbrushing which have been ascertained to be insufficient in the majority of adults and adolescents/children [3–6, 19]. Therefore, we undertook analyses to spot the reasons for the lack of efficacy of the *amabrush*® design. Possible deficiencies might be (1) poor bristle quality or (2) improper bristle alignment. Bristle quality was not investigated in the present study. We assessed the quantity/intimacy of bristle contact to the tooth surfaces during the cleaning procedure in casts of the probands’ upper and lower jaws. We found out that in none of the jaws (neither wide nor small; neither long nor short) the bristles stood in optimal contact to the teeth. The outer bristle row stood either in distance to the tooth surfaces or touched the palatal gums. Furthermore, the bristles are not aligned in a 45° angle against the longitudinal axis of the teeth (as postulated), but partially parallel to the tooth surfaces. The authors recommend a (re)evaluation of the bristle alignment towards the tooth surfaces. The bristles should be packed more densely and should oscillate more widely. Another problem might be the size of the mouthpiece. Owing to its relatively large width, it might adjoin to the ascending branch of the mandible and, thus, be deviated forwardly. Body or jaw size were not included in the in-/exclusion criteria to our study and thus haphazardly distributed in the study sample of ten males and ten females. Only one size of mouthpieces was delivered by Amabrush GmbH (Vienna, AT) as they provided us with a refined prototype of the mouthpiece. A choice of different sizes (and their assignment to users according to the configuration of their dental arches) might reduce misfit. The question is how many stock sizes are needed to cover most jaw lengths and widths. In partially edentulous dental arches, auto-cleaning devices seem to be not reasonably applicable at all. By the time this study was published, Amabrush GmbH (Vienna, AT) had gone out of business. In the light of our unfavorable results, we refrained from further test set-ups (e. g., a prolonged brushing time of 20 s using the auto-cleaning device, its long-term use, or the testing of other parameters than plaque removal) for the time being.
